# Lectures on Subjects Connected with Clinical Medicine

**Published:** 1836-07-01

**Authors:** 


### Lectures on Subjects connected with Clinical Medicine. By *P. M. Latham*, M.D. Octavo, pp. 322. Longman and Co. 1836.

Of all the errors, defects, and deceptions incident to medicine—(and this is taking a pretty considerable range)—there is not one more productive of mischief, misery, and false conclusions, than the want of a systematic and philosophic mode of investigating the nature and seats of disease through the medium of symptoms. We have made far greater advances in pathology than in semeiology. Our merest tyros can trace out the seats (more properly the *effects*) of diseases in the dead body, while nine-tenths of our veteran physicians go the wrong way to work, in ascertaining the said diseases in the living body !!

In general, the patient is allowed to tell a long tale, which is little to the purpose, or dwell on symptoms which are secondary or unessential, and then a few questions are asked, without order, method, or connexion—after which the guinea is paid, and the prescription is just as likely to do good as harm. Such is the routine, as is well known, in the metropolis and other great cities, where the turnpike-gates of physic are monopolized by a few FARMERS-GENERAL in our profession!

It is more than 30 years ago, that we were struck with the want of method so conspicuous in the investigation of diseases, as cognizable by symptoms in the living body. We then set about a plan for our own private use, and we verily believe that much of our professional success—such as it is—has resulted from the procedure then adopted, afterwards improved by practice. If we give an outline of that plan, we may, perhaps, confer some benefit on our *junior* brethren—for as to our *seniors*, it is too late for *them* to learn, even if they condescend to listen to any proposal from any qaarter.

In many of the acute and inflammatory diseases, it does not require a very minute or systematic plan of inquiry to ascertain the nature of the case. Encephalitis, pleuritis, peritonitis, cystitis, &c. present such striking features, that few can make any very serious mistakes, if they are at all grounded even in the rudiments of their profession. But nineteen-twentieths of the diseases or disorders which present themselves to the medical practitioner, in the existing state of civilization, are of a chronic character, and very difficult to ascertain. It is in these last that the value of *method* becomes conspicuous.

We shall not write a treatise on semeiology in this place, but give a short exposé of our own practice for more than a quarter of a century past. The disorders of organs, whose alterations of structure are not visible or tangible, can be only ascertained by an examination of their own peculiar *functions*, and the *sympathetic affections* which they induce in other organs or structures. We should *first* direct our attention to the *proper* functional disorders, and afterwards to the sympathetic. This order is very often reversed by practitioners—and the secondary effects of diseases attract more of their attention than the primary. Thus, for example, where giddiness, pain, or other affection of the head is complained of, the patient, at the same time, exhibiting symptoms of gastric or intestinal disorder, the cause is at once pronounced to be abdominal, which conclusion is very generally in accordance with the patient's opinions and prejudices. Hardly a day passes, without our witnessing the injurious consequences of this mode of jumping at conclusions.

Among the upper classes of life, we are obliged to listen to the patient's tale, though we generally cut it as short as possible, in order to get at our own plan of investigation. It is usually thus: beginning with the organ of mind, as well as the great nervous source and centre, we inquire first into its corporeal functions—the state of the senses, &c. Then we inquire into the intellectual powers, and determine whether they are impaired or perverted—diminished or excited.

The functions of the spinal marrow, as a continuation of the brain, are then run over, and they are easily determined by the state of sensation, motion, &c.

The functions of the ganglionic nerves are next to be looked to. Digestion, assimilation, respiration, secretion, and excretion. In this short survey of the chief vital processes, we rarely fail to detect the chief disorder, as connected with one or more of the functions examined. Then it is that we are to direct our investigation specifically to the particular disorder, after having detected other collateral affections to which the patient himself would not, otherwise, have drawn our attention. We were gratified to observe that Dr. Latham had been in the habit of pursuing a similar course, as proved by the following extract.

"The patient being placed before me, I ask him no question until I have learnt every thing worthy of remark which my own eyes can inform me of. His physiognomy; his complexion, whether florid, pale, or dusky; the general bulk of his body, whether large and full, or spare and wasted; the condition of particular regions, whether swelled or attenuated; and of the surface, whether there be any eruptions or sores upon it, and what is their character; and, lastly, the power of locomotion, whether he have free use of his limbs or not.

All these are most important particulars, and we ought to make much of them. There can be no doubt concerning them; they are objects of our own observation, and come to us authenticated by the testimony of our own senses. One step securely ascertained leads to another; and from what we see upon the exterior, we obtain a clue for directing our inquiry to the seat and centre of the disease within. If locomotion be hindered, we look well to the brain and spinal marrow; if there be the livid lip and dusky skin, we scrutinize particularly the condition of the heart and lungs; if the whole body, or some of its parts, be attenuated, we examine well the organs of nutrition.

Having thus learnt all I can with my own eyes, and felt the pulse and seen the tongue, I next proceed, in taking the case, to that further inquiry in which the patient takes a part: and, first, I ask him concerning his general sensations, especially whether he be hot or cold; and I endeavour to learn whether his heat and cold occur under conditions which constitute fever.

Next, I enquire into the state of particular organs; and, beginning with the head, I ask after pain, vertigo, and sense of weight, the sight and the hearing, and sleep and wakefulness. Many of these things are only glanced at, or perhaps passed over altogether, if there be no reason to suspect disease of the brain.

Then, passing to the chest and respiratory organs, I ask concerning pain and cough and expectoration, and the state of the breathing under various conditions of exertion and in different postures of the body; and I learn the force and extent of the heart's pulsation.

These things are hardly dwelt upon, or soon despatched, if there be no suspicion of disease in the chest; but if there be, not all we can learn by simple inquiry is enough to ascertain its nature. The patient must, moreover, be submitted to the process of auscultation. This process, however, in order to avoid interruption, I postpone until other inquiries are finished.

Lastly, proceeding to the abdomen, I ask here also concerning pain and uncomfortable sensations, the appetite, the digestion, and the evacuations, their frequency, quantity, and appearance; and then I ascertain with my hand its form and fulness, the possible enlargement of particular viscera, the effusion of fluid, or the existence of pain upon pressure.

Here the examination of the patient ceases, as to his actual condition; but the history of his complaint remains to be learnt, its origin and its progress hitherto, and its probable exciting cause." 50.

There is more than one advantage in this systematic—we would say scientific—mode of procedure. It secures to the physician the best data for ascertaining the nature of the disease—it impresses the patient with a favourable opinion of the skill and science of the practitioner. A careless and *off-hand* mode of divining, rather than of investigating diseases, may succeed in the hands of an Abernethy and a few others; but it will not generally answer the purpose. And, after all, it is the greatest stigma on the talents and judgment of illustrious individuals. They abused their powers and their reputation, and we have no hesitation in saying, this same *off-hand* routine has sent thousands to a premature grave. We are perfectly indifferent to the anger which this sentiment may engender in the disciples or admirers of the illustrious dead or living. We are conscious of its truth—and we care not what interpretations may be put upon our motives.

Nothing can be more true than the following remark; but we beg to observe that the precept can only be put fully into practice under two particular circumstances:—First, in hospital practice, where the physician can *dictate* to the patient; and, secondly, where the established reputation of the consultant enables him to *dictate* to the private patient, whatever be his rank.

"Perhaps it would seem more in the order of nature, and therefore the best method, to take the history first of all. Formerly I used to do so, but I found it practically inconvenient. If you first learn the existing complaint, you know how much of its previous history you will require to illustrate it; but if you first inquire the history, since you do not yet know what it is to illustrate, you cannot tell how much of it you shall want, and must allow the patient to tell what he thinks fit; and, since every person's complaint is interesting to himself, he is apt to discourse about it rather too much at large, and too little to edification. Therefore it is, that I now always inquire the history last, inverting (if you please) the order of nature; and I take care *to make the patient answer express questions rather than leave him to expatiate at his own discretion.*" 50.

This is good advice, and such as we have long acted on in private practice.

The following passage, too, deserves serious reflection.

"I have always thought that hospitals are not converted to half the good they are calculated to serve as schools of medicine; and I think so still.

I have always thought that, in hospitals, knowledge is perpetually running to waste for want of labourers to gather it; and I think so still.

I have always thought that, in our schools, every mode of lecturing has been unduly exalted above clinical lecturing; and every place where knowledge is to be had, or is supposed to be had, has been unduly preferred to the bed-side; and I continue to think thus.

With respect to clinical lecturing itself, custom has robbed it of its peculiar character, and, withal, of half its advantages and half its popularity. It has been separated too much from the wards and the bedside, and has deviated into a discussion of abstract pathology and therapeutics. There may, indeed, be things which can be discussed with convenience and propriety only apart from the patient; and so let them be: but these bear a small proportion to the multitude of things which can only be learnt at his bedside, and in his very presence.

Here is a hospital containing 500 patients—a wonderful spectacle! Hither resort hundreds of students from every part of the Empire. Here they see what the majority will never see again, after the period of their pupilage is over.

They see collected in one place every variety of disease, and every variety of injury, and numerous specimens of each. What an opportunity of instruction gained, if rightly used; what an opportunity lost, if neglected!

And which is generally the case? Is the opportunity, in fact, generally used or neglected? I speak from my own certain conviction, and I answer, that it is generally neglected. I know that five out of six of those who profess to attend the medical practice of this hospital (and it is the same at other hospitals) never watch a single case of disease through its entire course, during the whole period of their pupilage. I say this with great sorrow, and as a warning to those whose pupilage has yet to begin. This is what I mean by the materials of knowledge running to waste." 31.

The truth of this statement has long been felt and acknowledged. The CHIRURGO-MANIA is as universal at all times as the influenza was in 1834. Every student in London *considers* that he is to be a SURGEON—and every one afterwards *finds* that he is a physician—in nineteen cases out of twenty. What are the consequences? The young practitioner is perpetually embarrassed with the treatment of diseases which he neglected to observe on the great theatre of the London Hospital, whilst his purely surgical knowledge is a granary from which he can draw no grain!

Our author thinks, and justly, that one clinical lecture is worth 50 of those starch systematic sermons delivered to drowsy benches, on the theory and practice of medicine. The fact is, that the curriculum of education is too complicated, and the time too short. There ought to be four or five years dedicated to study before practice is entered upon, in private life.

"Then there are lectures upon botany; lectures upon midwifery, and upon the diseases of women and children; lectures upon forensic medicine. Now, I dare not say that the subject-matter of all these lectures is not of the highest order; and therefore I must not tell the student that *this* knowledge is less important than *that*, and that one lecture he may attend less diligently than another; I must only speak generally upon so delicate a subject, and contrive to intimate my opinion without giving offence. The prudent householder, when he would furnish himself a house, sees well enough that some things are of mahogany, and some of rosewood, and others of ebony and gold. He sees much for beauty, and much for use, and longs, perhaps to possess all. But his question is, 'Can I afford to possess them?' and his answer, 'No, not at present; and I must wait until I can.' So, when there is laid before the student this magnificent furniture of the mind, and he asks himself, 'Can I possess it?' I will answer the question for him, 'No, you cannot at present.'—'But can I ever possess it?'—'Certainly you can.'—'But how?'—'By diligence and by *time*. Your studies will not be limited to the period of your pupilage, and you will know all these things in *time;* but certainly not in the brief space of two or three years.'" 35.

There is a fund of good advice to students in this book; but the principal portion of it is occupied with auscultation. Dr. Latham has simplified the study of this important indication, and properly condemned those minute refinements which are puzzling even the authors of them every day. We shall conclude with one more extract.

"I have met with a frightful affection in children; but what its nature was I could never tell, until auscultation enabled me to unravel it. It commonly passes for inflammation of the lungs. But, when children have got well, they have got well so soon and so entirely, that I could never believe the disease to be pneumonia, although the symptoms seemed to indicate that it could be nothing else.

Last summer I went out of town to see a little boy, seven or eight years of age, whose life was very precious to his family. He was thought to be dying of inflammation of the lungs. I found him raised up in bed, supported by his nurse, and breathing with all his might. His skin was hot; his face flushed; and his chest heaved, and his nostrils quivered frightfully. There was no croupy sound. Whatever the disease was, it was all within the chest. I percussed the chest; it sounded well in every part. I listened: the air entered freely, and reached every cell and vesicle of the lungs; but there was not the least perception of the natural respiratory murmur: a shrill Sibilus had taken place of it altogether. Wherever you applied your ear to the chest, you might fancy you heard the piping and screaming of a nestful of unfledged birds.

But what was this disease? Surely it was inflammation, largely diffused over the mucous surface throughout the bronchial ramifications, but inflammation as yet only in its *first* stage; for the air, as it passed through them, did not mingle with a particle of fluid any where, and the sound it produced was a dry Sibilus only.

But *how* inflammation yet only in its *first* stage? The boy had been already ill four days. Still it might be inflammation in its *first* stage. The boy continued ill two days longer, with the same kind and the same degree of suffering; and then, under the influence of tartar emetic, the fever began gradually to subside, and the dyspnœa to abate. The Sibilus gradually gave way to the healthy respiratory murmur, and he was well again *without expectoration of any kind.* The inflammation began and ended with the *first* stage; and, although it continued with great severity for a week, it never got beyond the *first* stage.

This is an instance which strikingly shows the value of Auscultation in detecting at once the state of things, about which you might go on conjecturing and conjecturing for ever what it *possibly* might be, and not gain the least assurance what it *actually* was.

In adults sometimes, but not so frequently as in children, I have met with the same evidences of acute inflammation widely diffused through the bronchial ramifications, and remaining in this its first stage for days and days together. In the mean time, their mucous surface has still been dry throughout a great part of both lungs, and the ear has continued for days and days together to hear no other unnatural sound but a Sibilus. Convalescence has taken place without expectoration, and the Sibilus has given way, without the intervention of any *moist* sound, at once to the murmur of health.

But such inflammation, after lingering long in the first stage, will sometimes pass beyond it; and the whole mucous surface, that was previously dry, will pour forth an enormous secretion, and the widely diffused Sibilus will be changed into a widely diffused Crepitation. Still the lungs are unhurt beyond the lining membrane of the air-passages, and the patient will get well, if he be not suffocated by the enormous expectoration.

I am speaking of a disease which must be distinguished from asthma, according to the usual acceptation—a disease not habitual to the individual, and of which, perhaps, he has never suffered a previous attack. I am speaking of acute inflammation extending throughout the bronchial ramifications, and reaching, perhaps, the vesicular structure of the lungs, putting on a peculiar form, and affecting a peculiar course; but still of acute inflammation, as further evidenced by the remedies necessary for its relief.

During the last summer I saw a gentleman, who had been, two days previously, seized rather suddenly with feverish symptoms, and with the most dreadful dyspnœa. His lips were blue; he was labouring for breath, and coughing with hard and ineffectual efforts to rid himself of something which seemed to tease the larynx, but no expectoration followed.

Cupping on various parts of the chest (the state of vascular action required that blood should be drawn), and tartar emetic in frequent doses, were the remedies employed; but in the same state of agony he remained for a week, propped up in bed, striving with all his might to free himself from his oppression, coughing and endeavouring to expectorate, but ineffectually.

What was going on all this time? There was anguish enough for any disease of the most formidable name; for fluid in the pericardium; for extensive hydrothorax; for induration of a whole lung; for stricture at some orifice of the heart. A few years ago, the most sagacious physician could only have guessed at the real state of disease, and probably would have guessed wrong. Such severe dyspnœa, so long continued, without expectoration, would probably have determined his diagnosis to hydrothorax.

But what was the disease? Every part of the chest sounded well to percussion. The heart beat regularly, and with a natural sound, only with too great frequency.

What could it be? There reached the ear from every part of the chest to which it was applied a loud Sibilus. The disease was an inflammation largely diffused through all, perhaps, of the bronchial passages, great and small; inflammation abiding long in its first stage, and limiting itself to one structure.

But in this case the inflammation ultimately passed beyond its first stage; for ultimately there arose an immense expectoration, and so the disease reached a favorable termination." 196.

We regret to observe some ill-natured remarks on certain publications, given as advice to students.

"Mind (says Dr. L.) that you are not betrayed to commit yourselves unwarily to books (especially of modern date) upon *diet* and digestion, upon the liver and the stomach. The public is well understood to have such a relish for reading upon such subjects, that *new motives* have been thus let in for medical authorship, which are not very creditable. Any of *you*, who may feel himself a little sharp and clever, might write such a book to-morrow, with a tolerable chance of *all* its attendant advantages."

Now, as Dr. Latham would not expend powder and shot upon the herd of mere quacks, it is perfectly clear that the above passage is levelled at Paris, Philip, Abernethy, Abercrombie, and Johnson. We leave those above, who are still living, to defend themselves. A word or two in respect to the senior Editor. Dr. L. says to his pupils—" never read any book that bears internal marks of being addressed more to the public than to the profession. They are all bad, and many of them dishonest."

Dr. Johnson's work on Indigestion was *first* published in the 4th edition of his work on Tropical Climates, as Dr. L. may see—a work that surely could not be suspected of being addressed to the public—at least the public of this hemisphere. A few copies of that part were struck off for distribution among his friends; and many of these last advised a separate publication. The result was the sale of eight thousand copies. That Dr. Johnson's practice was increased by this work he does not deny—but that *practice* was not always accompanied by corresponding *emolument*. He can safely say that, in consequence of the said *bad and dishonest* book, he has been consulted by at least five hundred of his brethren on their own account, and without any honorarium. When Dr. Latham tells his sucking pupils that any of *them* might " write such a book to-morrow," he no doubt flattered their vanity, and he certainly is, in some measure, accountable for the swarm of publications that must emanate from the Bartholomew school forthwith—especially after the following piece of encouragement.

"There is not a medical publisher in this town, who would not give you (the sucking pupil) something handsome for a book 'UPON THE STOMACH.'"

Indeed! let them try. If any publisher gives such a pupil "*something handsome*"—or even credit for the paper of such a book, we know nothing of the "*trade.*"

But a more absurd and erroneous statement than the above, was never delivered to a group of gaping pupils by a grave hospital physician. If any such pupil attempted this easy task, *and succeeded,* it could be only by transcribing, *verbatim et literatim,* the work of some other person. Every *original* line that he wrote would be an original error—and if he tried *compilation,* he would be more likely to select the faults than the merits of the authors. But the whole thing is sheer nonsense, and looks far more like a little unguarded ebullition of jealousy, than of judgment.

